# Do we need biopsies of metastases for colorectal cancer patients?

**DOI:** 10.1038/sj.bjc.6605131

**Published:** 2009-07-07

**Authors:** I Floriani, D Santini, V Torri, C Cremolini, A Falcone, F Loupakis

**Affiliations:** 1 Dipartimento di Oncologia, Istituto di Ricerche Farmacologiche ‘Mario Negri’, Milano, Italy; 2 U.O. Oncologia Medica, Università Campus Biomedico, Roma, Italy; 3 U.O. Oncologia 2 Universitaria, Azienda Ospedaliero-Universitaria Pisana, Pisa, Italy

**Sir**

We read with great interest the paper by Molinari *et al* (2009), recently published in the *British Journal of Cancer*. The study compares findings in primary colorectal cancer and paired metastases in 38 patients, showing differences with respect to EGFR pathway deregulation, which may imply a different response to anti-EGFR monoclonal antibodies. As a consequence, the authors suggest that the analysis of metastatic lesions should be considered both for patient management and when planning clinical trials with anti-EGFR drugs.

Although this statement can be considered as a hypothesis tenable in principle, it seems not to be supported by the results provided by the study itself, for the following reasons:

1. Statistical analysis is lacking in terms of study question definition and sample size calculation and therefore all the considerations made are not supported by a formal hypothesis verification;
2. Comparisons are made in terms of concordance, using the kappa index, instead of exploiting discrepancies between the two paired sets of data. However, even looking at concordance indexes, kappa values were equal to 0.83, 1, 0.73, 1, 0.49 for *KRAS* mutational status, *BRAF* mutational status, PTEN protein expression, EGFR protein expression and *EGFR* gene status, respectively, showing that only for *EGFR* gene status the concordance did not reach a ‘good’ value. As no intra-sample concordance results are available, the question whether these differences are because of a true discordance between primary tumour and metastases or rather due to an intra-sample variability (i.e., variability (a) between different areas within the same sample because of biological heterogeneity (Al-Mulla *et al*, 1998) or (b) to technical reproducibility) remains unanswered.
3. Looking at Table 2,
  1. for *KRAS* the data show two patients mutated in primary tumour whose result became wild type in distant metastatic sites, whereas one patient wild type in primary tumour became mutated;
  2. only for three patients with PTEN-negative metastases but positive primary tumours, the finding of non-response is consistent with biological hypothesis and other retrospective proof of principles (Loupakis *et al*, 2009);
  3. for *BRAF* no modification was seen;
  4. full concordance was detected for EGFR IHC analysis, whereas for *EGFR* gene status the problems related to the reproducibility are well known.

Even looking at these data in a ‘qualitative’ way, the overall picture does not seem to suggest a clear trend towards a negative predictive effect of metastases, and only for PTEN there is some evidence towards a negative effect.

In the clinical practice, the biopsy of the metastatic lesion could be an invasive procedure, not free from risks and causes delays in treatment start, other than understandable anxiety for the patient. Therefore, costs and advantages should be well balanced. Moreover, it should be considered (1) that the only universally accepted determinant of resistance to anti-EGFR MoAbs are *KRAS* mutations, (2) that *post hoc* analyses of randomised studies, which led to the regulatory restriction for anti-EGFR MoAbs to *KRAS* wild-type patients were conducted almost exclusively on primary tumours (Amado *et al*, 2008; Karapetis *et al*, 2008) and (3) that other series greater than the present found a degree of correlation between primary tumours and related metastases in terms of *KRAS* mutational status that does not justify, at present, the clinical need for biopsies of metastases (Artale *et al*, 2008; Santini *et al*, 2008; Loupakis *et al*, 2009). Given the retrospective nature of the study, which exposes the results to selection and verification biases, and given the small sample size, we do think that no definitive clinical implication can be driven from this study. Rather, it is important to stress the need for prospective, properly powered studies, aimed to evaluate the importance of tumoral sampling at time of treatment's start for the molecular prediction of benefit from anti-EGFR MoAbs, but, at the same time, there are no data for supporting the need for biopsies of metastases in the routinary practice.
